# SLURP-1 Controls Growth and Migration of Lung Adenocarcinoma Cells, Forming a Complex With α7-nAChR and PDGFR/EGFR Heterodimer

**DOI:** 10.3389/fcell.2021.739391

**Published:** 2021-09-14

**Authors:** Maxim L. Bychkov, Mikhail A. Shulepko, Olga V. Shlepova, Dmitrii S. Kulbatskii, Irina A. Chulina, Alexander S. Paramonov, Ludmila K. Baidakova, Viatcheslav N. Azev, Sergey G. Koshelev, Mikhail P. Kirpichnikov, Zakhar O. Shenkarev, Ekaterina N. Lyukmanova

**Affiliations:** ^1^Bioengineering Department, Shemyakin-Ovchinnikov Institute of Bioorganic Chemistry RAS, Moscow, Russia; ^2^Phystech School of Biological and Medical Physics, Moscow Institute of Physics and Technology, Dolgoprudny, Russia; ^3^Group of Peptide Chemistry, Branch of Shemyakin-Ovchinnikov Institute of Bioorganic Chemistry RAS, Pushchino, Russia; ^4^Department of Structural Biology, Shemyakin-Ovchinnikov Institute of Bioorganic Chemistry RAS, Moscow, Russia; ^5^Department of Molecular Neurobiology, Shemyakin-Ovchinnikov Institute of Bioorganic Chemistry RAS, Moscow, Russia; ^6^Biological Faculty, Lomonosov Moscow State University, Moscow, Russia

**Keywords:** lung cancer, intracellular signaling, Ly6/uPAR, nicotinic acetylcholine receptor, SLURP-1, A549, Lynx1

## Abstract

Secreted Ly6/uPAR-related protein 1 (SLURP-1) is a secreted Ly6/uPAR protein that negatively modulates the nicotinic acetylcholine receptor of α7 type (α7-nAChR), participating in control of cancer cell growth. Previously we showed, that a recombinant analogue of human SLURP-1 (rSLURP-1) diminishes the lung adenocarcinoma A549 cell proliferation and abolishes the nicotine-induced growth stimulation. Here, using multiplex immunoassay, we demonstrated a decrease in PTEN and mammalian target of rapamycin (mTOR) kinase phosphorylation in A549 cells upon the rSLURP-1 treatment pointing on down-regulation of the PI3K/AKT/mTOR signaling pathway. Decreased phosphorylation of the platelet-derived growth factor receptor type β (PDGFRβ) and arrest of the A549 cell cycle in the S and G2/M phases without apoptosis induction was also observed. Using a scratch migration assay, inhibition of A549 cell migration under the rSLURP-1 treatment was found. Affinity extraction demonstrated that rSLURP-1 in A549 cells forms a complex not only with α7-nAChR, but also with PDGFRα and epidermal growth factor receptor (EGFR), which are known to be involved in regulation of cancer cell growth and migration and are able to form a heterodimer. Knock-down of the genes encoding α7-nAChR, PDGFRα, and EGFR confirmed the involvement of these receptors in the anti-migration effect of SLURP-1. Thus, SLURP-1 can target the α7-nAChR complexes with PDGFRα and EGFR in the membrane of epithelial cells. Using chimeric proteins with grafted SLURP-1 loops we demonstrated that loop I is the principal active site responsible for the SLURP-1 interaction with α7-nAChR and its antiproliferative effect. Synthetic peptide mimicking the loop I cyclized by a disulfide bond inhibited ACh-evoked current at α7-nAChR, as well as A549 cell proliferation and migration. This synthetic peptide represents a promising prototype of new antitumor drug with the properties close to that of the native SLURP-1 protein.

## Introduction

Nicotinic acetylcholine receptors (nAChRs) are ligand-gated ion channels that are activated by acetylcholine (ACh) and are involved in regulation of many vital processes in the central and peripheral nervous system, including synaptic transmission and plasticity, neuronal plasticity, memory, cognition, addictive behavior, pain transmission and muscle contraction (Feduccia et al., 2012; Koukouli and Maskos, 2015; Zoli et al., 2018). Recently, however, a lot of data on the expression of nAChRs in non-neuronal cells, and on their participation in regulation of epithelial cell homeostasis and the immune system were published (Wessler and Kirkpatrick, 2009; Kulbatskii et al., 2018; Zoli et al., 2018). Nicotinic acetylcholine receptor of α7 type (α7-nAChR) is one of the most widespread type of nAChRs expressed in epithelial and immune cells (Zoli et al., 2018). The non-neuronal α7-nAChR signaling is implicated in the regulation of inflammation (de Jonge and Ulloa, 2007), terminal epithelial homeostasis (Grando, 2006), vascularization (Heeschen et al., 2002), and even in cytokine secretion during COVID-19 infection (Kashyap et al., 2020) and regulation of expression of ACE2,–the SARS-CoV-2 entry receptor (Leung et al., 2020; Russo et al., 2020).

There are also strong evidences of α7-nAChR involvement in oncogenic transformation and tumor progression upon activation by nicotine (Grando, 2014; Schaal and Chellappan, 2014). Nicotine and tobacco nitrosamines [4-(methylnitrosamino)-1-(3-pyridyl)-1-butanone (NNK) and N-nitrosonornicotine (NNN)] contained in tobacco smoke cause oncogenic transformation of normal cells (Arredondo et al., 2006a) and drive proliferation, migration, and invasion of breast, pancreatic, and lung cancer cells (Dasgupta et al., 2009; Gankhuyag et al., 2017; Sarlak et al., 2020). Notably, nicotine and nitrosamines demonstrate greater affinity to the α7 receptor than endogenous ACh (Grando, 2014). In lung cancer cells the pro-oncogenic effect of nicotine can be further enhanced by upregulation of the α7-nAChR expression in response to the nAChR activation by nicotine (Schuller, 2012). The nicotine signaling in lung cancer cells can also be enhanced by mitogenic receptor tyrosine kinases (RTKs) activation due to formation of heterocomplexes between α7-nAChRs and some RTKs, such as epidermal growth factor receptor (EGFR) (Chernyavsky et al., 2015). Along with the cell membrane, α7-nAChRs are also located on the mitochondrial membrane, where they can inhibit a mitochondrial membrane pore formation and apoptosis induction (Gergalova et al., 2012; Kalashnyk et al., 2012).

Activation of cell-surface α7-nAChR can lead to two main types of response: ionotropic, associated with the Ca^2+^ influx into the cytosol; and metabotropic, which activates mitogenic intracellular signaling pathways without opening of the nAChR channel (Grando, 2014). The protein kinase C (PKC), mitogen-activated protein kinase (MEK)/extracellular signal-regulated kinase (ERK), and phosphoinositide 3-kinase (PI3K)/protein kinase B (AKT)/mammalian target of rapamycin (mTOR) pathways are the main intracellular mediators of the α7-nAChR signaling responsible for growth, migration, and invasion of lung cancer cells (Tsurutani et al., 2005; Arredondo et al., 2006b; Carlisle et al., 2007; Chernyavsky et al., 2009; Davis et al., 2009; Mucchietto et al., 2018). In keratinocytes, the α7-nAChR activation upregulates some transcriptional factors e.g., signal transducer and activator of transcription 3 (STAT3), GATA-binding factor 2 (GATA2) (Arredondo et al., 2007b), and NFκB (Chernyavsky et al., 2010); so, the dysregulation of α7-nAChR can also lead to loss of control over gene expression and drive the progression of epithelial cancers. Activation of the PI3K/AKT/mTOR pathway by α7-nAChR can inhibit the cell senescence and apoptosis resulting in the chemotherapy resistance (Dasgupta et al., 2006; Junhui et al., 2009). In addition, the α7-nAChR activation can promote angiogenesis (Brown et al., 2012) and inhibit immunosurveillance (Qiu et al., 2004). On the other hand, the activation of α3-containing nAChR by nicotine mediates remodeling of the extracellular environment (Arredondo et al., 2003), creating a microenvironment favorable for migration and invasion. Thus, nAChRs and particularly the α7 receptors are involved in all stages of epithelial cancer progression; and inhibition of α7-nAChRs may be a promising strategy for lung cancer therapy.

There are several endogenous three-finger proteins belonging to the lymphocyte antigen 6/urokinase plasminogen activator receptor (Ly6/uPAR) family that negatively modulate nAChRs without complete inhibition and have the promising properties for cancer therapy. One of such proteins, the human secreted Ly6/uPAR-related protein 1 (SLURP-1), was initially described as a paracrine regulator of keratinocyte homeostasis. Point mutations in the *SLURP1* gene lead to the development of skin disease, palmoplantar keratoderma Mal de Meleda (Arredondo et al., 2005; Perez and Khachemoune, 2016). SLURP-1 has a rather flexible spatial structure (Paramonov et al., 2020), and site-directed mutagenesis suggested the possibility of its simultaneous interaction with several target receptors, by means of three elongated and conformationally mobile loops, and a β-structural core (“head”) of the protein (Shulepko et al., 2021). SLURP-1 interacts with α7-nAChRs (Chernyavsky et al., 2015; Lyukmanova et al., 2016a), induces keratinocyte apoptosis (Arredondo et al., 2005), and protects the oral keratinocytes from oncogenic transformation by tobacco-derived nitrosamines (Arredondo et al., 2007a; Kalantari-Dehaghi et al., 2012). SLURP-1 expression is down-regulated in primary and metastatic melanomas compared with normal cells (Bergqvist et al., 2018; Arousse et al., 2019), moreover the elevated level of SLURP-1 in plasma correlates with a better survival prognosis for pancreatic cancer patients (Throm et al., 2018). Thus, SLURP-1 can be considered a prototype antitumor drug, but its effect on cancer and normal cells, its targets and active centers should be studied in details.

Previously we have shown that recombinant analogue of human SLURP-1 (rSLURP-1) selectively inhibits ACh-evoked currents through α7-nAChR (Lyukmanova et al., 2016a) and suppresses the growth of different carcinoma cells (Lyukmanova et al., 2014, 2018; Shulepko et al., 2020a). The recombinant protein also suppresses the nicotine-induced lung cancer cell proliferation via interaction with α7-nAChR (Shulepko et al., 2020b). The PI3K/AKT/mTOR and inositol 1,4,5-trisphosphate (IP3) pathways are probably involved in the antiproliferative activity of rSLURP-1 in lung adenocarcinoma A549 cells (Shulepko et al., 2020b). In the present study, we further investigated the rSLURP-1 effects in A549 cells, determined the intracellular pathways involved in its action, revealed its new non-cholinergic molecular targets, and identified the primary active site responsible for the SLURP-1 antitumor activity.

## Materials and Methods

### Materials

Genes of the chimeric proteins NTII/SL-1(I), NTII/SL-1(II), NTII/SL-1(III), and SL-1/NTII(I) were obtained by site-directed mutagenesis on the basis of *pET22b/STII/NTII* and *pET22b/SLURP-1* expression plasmids, respectively (Lyukmanova et al., 2007; Shulepko et al., 2013). rSLURP-1 and chimeric protein SL-1/NTII(I) were isolated and refolded from *Escherichia coli* inclusion bodies as described previously (Shulepko et al., 2013). Chimeric proteins NTII/SL-1(I), NTII/SL-1(II), and NTII/SL-1(III) were produced in *E. coli* according to the protocols described in Lyukmanova et al. (2007). The purity and homogeneity of the protein preparations was confirmed by HPLC, MALDI-MS, and SDS-PAGE. Disulfide bond formation was confirmed in the reaction with Ellman’s reagent (Sigma-Aldrich, United States). The correct folding of the recombinant proteins was confirmed by 1D ^1^H NMR spectroscopy.

### Protein Phosphorylation Analysis

Phosphorylation of cellular signaling proteins was analyzed using the Bio-Plex magnetic beads assay with Bio-Plex Pro cell signaling reagent kit (Bio-Rad). Cells were incubated for 48 h with 1 μM rSLURP-1 added from 100% DMSO stock (protein stock concentration 250 μM) and lysed using provided buffer. Analysis was performed on Bio-Plex 200 machine (Bio-Rad) according to the manufacturer instructions using the Bio-Plex Manager 6.2 software (Bio-Rad).

### Cell Cultivation and Viability Assay

Human lung adenocarcinoma A549 cells (ATCC, United States) were grown (37°C, 5% CO_2_) in a DME medium with phenol red (PanEco, Russia), 10% fetal calf serum (Thermo Fisher Scientific, United States) and 2 mM L-glutamine (PanEco), abbreviated below as the A549 complete medium. Mouse primary lung fibroblasts were isolated from the lung of mouse embryos (the study was approved by IBCH RAS IACUC, protocol #312 from 21 December 2020) according to the previously described procedure (Edelman and Redente, 2018). Briefly, mouse embryos were isolated from female mice after cervical dislocation. The lung was removed from the thoracic cavity, placed into a 100 mm tissue culture petri dish, and cut into small pieces using a razor blade or surgical scissors. Then, 1 mL of StemPro Accutase Cell Dissociation Reagent (Thermo Fisher Scientific, United States) was added to the chopped lung and incubated at 37°C for 30 min. Then, the tissue was washed twice in HBSS and incubated at 37°C for 20 min in 0.5 mL of 0.25% Trypsin-EDTA. After incubation the cells were centrifuged for 5 min at 1000 × *g* and resuspended in fibroblast complete medium which was the A549 complete medium supplemented with Pen-Strep (10,000 U/mL penicillin, 10,000 U/mL streptomycin). Primary fibroblast cells were grown at 37°C and 5% CO_2_. All cells were subcultured at least twice a week.

To study an influence of the peptides or proteins on the A549 or primary fibroblasts cell growth, the cells were seeded in 96-well cell culture plates in the corresponding complete medium (0.5 × 10^4^ cells/well) and grown for 24 h. Thereafter rSLURP-1 from 250 μM DMSO stock or peptides from 10 mM DMSO stock solutions were diluted in the cell medium and added to the cells for further incubation during 48 h. The maximal DMSO concentration did not exceed 0.5%. The added DMSO did not influence the cell growth as was established in the additional experiments.

To analyze cell viability, we used water soluble tetrazolium salt 1 (WST-1) colorimetric test as described earlier (Lyukmanova et al., 2016a). Briefly, WST-1 (Santa Cruz, Dallas, TX, United States) and 1-m-PMS (1-methoxy-5-methylphenazinium methyl sulfate, Santa Cruz) were added to the cells in concentrations of 0.25 mM and 5 μM, respectively, for 1 h, and formation of colored product was measured at 450 nm with background subtraction at 655 nm on microplate reader. The data were normalized to averaged read-out from the control wells containing cells without added compounds.

### Knock-Down of *CHRNA7*, *EGFR*, and *PDGFRA* Genes in A549 Cells

To block expression of native α7-nAChR, EGFR, and platelet-derived growth factor receptor type α (PDGFRα), A549 cells were transfected with siRNA (Supplementary Table 1, Synthol, Russia). Cells were seeded in T25 cell culture flasks (1 × 10^5^ cells per well) and grown for 24 h. Then four different siRNA were mixed (1 μg per well), the mixture was diluted in the 100 μl transfection buffer (Pan-Biotech, Germany), incubated for 5 min and mixed with 15 μl of pre-diluted PanFect A-plus transfection reagent (Pan-Biotech, Germany). The final mixture was incubated for 30 min and added to A549 cells. The cells were incubated in CO_2_-incubator during 4 h and the cell media was replaced by the fresh one. After 48 h incubation, the cells were detached by Versene solution and divided into two parts. The first part was incubated with TRITC-labeled α-Bgtx (Sigma-Aldrich, T0195) for detection of expression of functional α7-nAChRs on the cell membrane. For EGFR and PDGFR detection, cells were incubated with primary antibodies (Abs) (sc-373746, Santa Cruz, for EGFR detection and ABIN5611263, Antibodies online, for PDGFRα) and with secondary TRITC-conjugated Abs (615-025-214, Jackson Immunoresearch). Expression of surface receptors was analyzed by flow cytometry. The second part of the cells was seeded in 96-well culture plates (50 × 10^4^ cells per well) and wound-healing assay was performed as described below.

### Wound Healing (Scratch) Assay

The *in vitro* wound healing (scratch) assay was performed as described elsewhere (Varankar and Bapat, 2018) with some changes. In brief, A549 cells and primary fibroblasts were seeded in 96-well cell culture plates in the corresponding complete medium (5 × 10^4^ cells/well) and grown for 24 h. Then the media from the wells was changed to serum-free media to minimize cell proliferation. After 8 h the wells were scratched with a sterile 10 μl pipette tip. Then, the cells were washed with PBS and treated with rSLURP-1 or peptide for 48 h. Pictures were analyzed after 0 and 24 h at 20× magnification at CloneSelect Imager (Molecular Devices, United States). The center of the plate was marked as a central reference point to ensure recording of the same area during the time course. Digital images were taken, and the scratch area was quantified using ImageJ (NIH, United States) and MS Excel software by measurement % of scratch surface, occupied by migrating cells. In each experiment, the duplicate measurements have been averaged.

### Cell Cycle Arrest in A549 Cells

Cells were seeded in T25 Cell Culture Flask (5 × 10^5^ cells/flask) and incubated with 1 μM rSLURP-1 for 48 h. Then the cells were detached from the flasks by trypsin, washed with EBSS, and fixed in ice-cold 70% ethanol for 4 h. After fixation, the cells were washed twice by EBSS, and DNA was extracted by 5 min incubation with the DNA extraction buffer (200 mM Na_2_HPO_4_ with 0.004% Triton X-100, pH 7.8). Then the cells were washed by EBSS, resuspended in the DNA staining solution (EBSS, 50 mg/ml propidium iodide, 0.2 mg/ml DNase free RNAse), and analyzed by Cytoflex flow cytometer (Beckman Coulter, United States). Data were analyzed by ModFit LT software (Verity House, United States).

### Study of Apoptosis in A549 Cells

To investigate apoptosis in A549 cells, we used Annexin V for detection of phosphatidylserine externalization,–one of the early apoptosis markers. Briefly, A549 cells were seeded on a 35-mm Petri dish (1 × 10^5^ cells/dish) and incubated with 1 μM rSLURP-1 for 48 h. After incubation, the cells were detached by the Versene solution and washed in annexin-binding buffer (V13246, Thermo Fisher Scientific). Then, the cells were incubated with Annexin V conjugated to Alexa 488 (A13201 Thermo Fisher Scientific) for 20 min, washed by annexin-binding buffer, and were analyzed on Cytoflex flow cytometer (Beckman Coulter, United States). Data were analyzed by CytExpert 2.4 software (Beckman-Coulter, United States).

### Affinity Purification and Western Blotting

Recombinant analogue of human SLURP-1 (1 mg/ml) was coupled to NHS-activated Sepharose 4 Fast Flow resin (Cat# 17-0906-01, GE Healthcare) according to the manufacturer’s manual. The empty resin blocked by 500 mM ethanolamine was used as a negative control. The membrane fraction of A549 cells (5 × 10^7^ cells per sample) was solubilized in 2% Triton X-100 (Cat# A4975, Panreac), diluted 10 times with TBS buffer [100 mM Tris (141940.0914, Panreac), 150 mM NaCl (141659, Panreac), pH 8.0], and incubated with the resin for 1 h in TBS. After that, unspecific bound proteins were sequentially washed out from the resin with five volumes of TBS, five volumes of TBS + 1 M NaCl + 0.5% Triton X-100, and five volumes TBS + 0.5% Triton X-100. The specifically bound proteins were eluted by five volumes of 200 mM Glycine (131340, Panreac) (pH 2.6) into non-reducing PAGE loading buffer for detection of EGFR and in reducing PAGE buffer for detection of α7-nAChR and PDGFRα. Western blotting was used to detect α7-nAChR (primary Abs ABIN5611363, Antibodies Online, 1:1000 and secondary Abs 111-035-003, Jackson Immunoresearch, 1:5000), EGFR (primary Abs sc-120, Santa Cruz, 1:1000 and secondary Abs 715-035-150, Jackson Immunoresearch, 1:5000) and PDGFRα (primary Abs ABIN5611263, Antibodies Online, 1:1000 and secondary Abs 715-035-150, Jackson Immunoresearch, 1:5000).

### Real Time PCR for miRNA Detection

Total mRNA from the cultured cells was extracted by the Aurum Total RNA Mini Kit (Bio-Rad) according to the manufacturer’s instructions. Total cDNA was synthesized using the Mint revertase (Evrogen, Russia) with miRNA-specific stem-loop primer. After that, real-time PCR was performed with the primers described in the Supplementary Table 1, and ready-to-use qPCR mix with the SYBR Green I fluorescent dye (Evrogen). Negative controls contained all the components of the PCR mixture but with cDNA replaced by mRNA gave no signal. All PCR reactions were performed using Roche Light cycler 96 real-time detection thermal cycler (Roche, Switzerland). Data was analyzed by the ΔCt method (Livak and Schmittgen, 2001) using Light-Cycler 96 SW1.01 software (Roche). The expression level of the genes was normalized to the expression level of the housekeeping non-coding RNA U6.

### Design and Chemical Synthesis of Peptides Mimicking the SLURP-1 Loops

The amide forms of the peptides (“loop I”: VKAYTCKEPXTSASCRTITRA, X stands for norleucine (methionine bioisosteric replacement); “loop III”: GCVARDPDSIGAAHLIFCG) were obtained by chemical synthesis using the Fmoc/TBTU methodology (Chan and White, 2000), starting with aminomethyl polystyrene acylated with Rink amide-linker at an initial loading of 0.4 mmol/g. Standard protocols were followed during stepwise chain elongations, except that *N*-terminal amino-acids were introduced using the corresponding N_α_ -Boc derivatives of valine and glycine. After completing the chain elongation, the protecting groups and the linear peptides were removed from the support using a mixture of TFA-DCM-TIPS-H_2_O-anisole (95: 3.5: 0.5: 0.5: 0.5: 0.5, 30 ml) in an inert atmosphere. The reaction mixtures were filtered from the polymer, evaporated *in vacuo* at a temperature not exceeding 40°C to a quarter of the initial volume and slowly added to cold diethyl ether. The precipitates were then filtered off on a glass filter, washed with diethyl ether and dried in a vacuum desiccator over potassium hydroxide pillets and paraffin overnight.

The disulfide bridges were formed under slightly basic conditions (200 ml of 0.1 M ammonium bicarbonate solution per 70–100 mg of peptide sample). The disappearance of sulfhydryl groups was assessed by the quantitative Ellman’s test (Andreu et al., 1994). After 3–4 days, the reaction mixtures were evaporated in vacuum to a volume of 50–60 ml and lyophilized. After that, first crude HPLC purifications were achieved using Vaydac C18 (250 × 21.4) column with isocratic elution using aqueous acetonitrile (21% acetonitrile/0.1% TFA in water for “loop I” and 24% for “loop III,” 10 ml/min). The fractions collected were analyzed by mass-spectrometry and the appropriate fractions were lyophilized and additionally purified by HPLC using a Jupiter C4 chromatographic column (4.6 × 250, Phenomenex, acetonitrile gradient 20–25% in 0.1% TFA at a flow rate of 0.2 ml/min). The homogeneity and purity (>95%) of peptides were confirmed by HPLC, MALDI-MS, and ^1^H-NMR spectroscopy (Supplementary Figure 3).

### Electrophysiological Recordings From *X. laevis* Oocytes

For expression of human α7-nAChRs in *Xenopus* oocytes, the linearized plasmid was transcribed using the T7 mMessage-mMachine transcription kit (Thermo Fisher Scientific, Carlsbad, CA, United States). The harvesting of stage V–VI oocytes from anesthetized female *Xenopus laevis* frog was previously described (Peigneur et al., 2019). Oocytes were injected with 42 nL of cRNA at a concentration of 1 ng/nL using a micro-injector (WPI, United States). The oocytes were incubated in a solution containing (in mM): 96 NaCl, 2 KCl, 1.8 CaCl_2_, 2 MgCl_2_, and 5 HEPES, (pH 7.4), supplemented with 50 mg/L gentamycin sulfate.

Two-electrode voltage-clamp recordings were performed at room temperature (18–22°C) using a Geneclamp 500 amplifier (Molecular Devices^®^, Downingtown, PA, United States) controlled by a pClamp data acquisition system (Axon Instruments^®^, Union City, CA, United States). Whole-cell currents from oocytes were recorded 1–4 days after injection. Bath solution composition was (in mM): 96 NaCl, 2 KCl, 1.8 CaCl_2_, 2 MgCl_2_, and 5 HEPES (pH 7.4). Voltage and current electrodes were filled with 3 M KCl. Resistances of both electrodes were kept between 0.7 and 1.5 MΩ. During recordings, the oocytes were voltage-clamped at a holding potential of −70 mV and continuously superfused with solutions. α7-nAChRs currents were evoked by 100 ms pulses of 100 μM ACh at 2 mL/min with 1–2 min washout periods between applications. Data were sampled at a frequency of 100 Hz and low-pass filtered at 20 Hz by using a four-pole Bessel filter. Peak current amplitude was measured prior to and following the incubation with rSLURP-1 and peptides. Data were analyzed using pClamp Clampfit 10.0 (Molecular Devices^®^, Downingtown, PA, United States) and Origin 7.5 software (Originlab^®^, Northampton, MA, United States).

### Statistical Analysis and Curve Fitting

Data are presented as mean ± SEM. Sample numbers (n) are indicated in the figure legends. Statistical analysis was done using two-tailed *t*-test, one sample two-tailed *t*-test, or one-way ANOVA followed by a Dunnett’s *post hoc* test as indicated in the figure legends. Differences in the groups were considered statistically significant at *p* < 0.05. To assess the concentration-response relationships, normalized data points were fitted with the standard slope inhibition equation: y(%) = 100% × (1–1/[1 + EC_50_/[protein]], where y(%) is the amplitude of the protein-induced effect, EC_50_ is the protein concentration at half-maximal efficacy, [protein] is the protein concentration. Analysis was performed using the GraphPad Prism 6.0 software.

## Results

### rSLURP-1 Reduces PTEN, mTOR, and PDGFRβ Phosphorylation in A549 Cells

Recently, we have shown that nicotine stimulates growth of lung carcinoma A549 cells and down-regulates the expression of phosphatase and tensin homolog deleted on chromosome 10 (PTEN), while rSLURP-1 abolishes these negative effects of nicotine (Shulepko et al., 2020b). At the same time, rSLURP-1 by itself inhibits growth of A549 cells, and this effect depends on α7-nAChR, EGFR, and β-adrenergic receptor (Shulepko et al., 2020b). α7-nAChR and EGFR are both enhance the cancer cell proliferation via activation of the PI3K/AKT/mTOR signaling pathway, and PTEN is a major negative regulator of this pathway (Misiura et al., 2020; Witayateeraporn et al., 2020; Cochard et al., 2021). Therefore, we decided to study the effect of rSLURP-1 on the phosphorylation level of the certain components of this intracellular signaling pathway (AKT, PTEN, and mTOR) in A549 cells.

**FIGURE 1:**
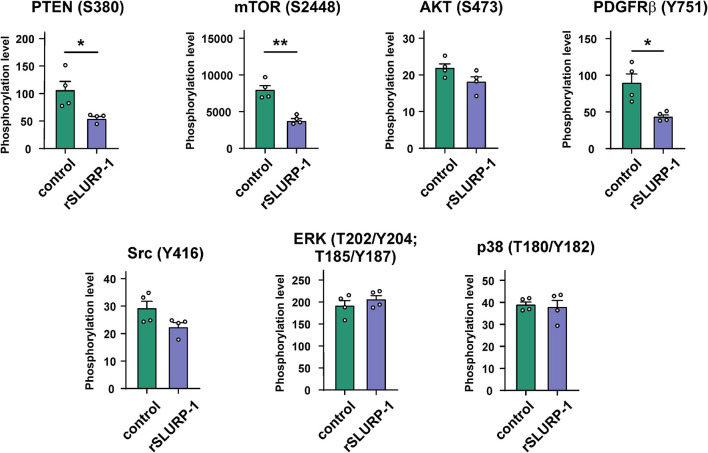
Effect of rSLURP-1 on phosphorylation of the Akt/mTOR and MAP/ERK pathways participants in A549 cells. A549 cells were incubated for 48 h with 1 μM of rSLURP-1; and PTEN (S380), AKT (S473), mTOR (S2448), PDGFRβ (Y751), Src (Y416), ERK (T202/Y204; T185/Y187), and p38 (T180/Y182) phosphorylation was detected by Bio-Plex magnetic beads assay. Data are presented as mean ± SEM, *n* = 4; * (*p* < 0.05) and ** (*p* < 0.01) indicate the significant difference by a two-tailed *t*-test.

The Bio-Plex magnetic immunoassay revealed that rSLURP-1 significantly inhibited PTEN phosphorylation at the S380 site, resulting in PTEN activation (Scully et al., 2014; Figure 1). In addition, we found that the rSLURP-1 application significantly reduced the mTOR phosphorylation (activator of the PI3K/AKT pathway) in A549 cells, thereby reducing its activity. Interestingly, we also observed a decrease in PDGFRβ phosphorylation at the Y751 site upon incubation with rSLURP-1 (Figure 1), which may lead to decrease in phosphorylation of PI3K (Kazlauskas and Cooper, 1990) and AKT (Zhang et al., 2015). Indeed, we found weak insignificant decrease of the AKT (S473) phosphorylation (Figure 1). The observed simultaneous PTEN activation and mTOR/PDGFRβ inactivation revealed down-regulation of the PI3K/AKT/mTOR signaling pathway in A549 cells upon incubation with rSLURP-1.

### rSLURP-1 Induces Cell Cycle Arrest but Not Apoptosis in A549 Cells

**FIGURE 2:**
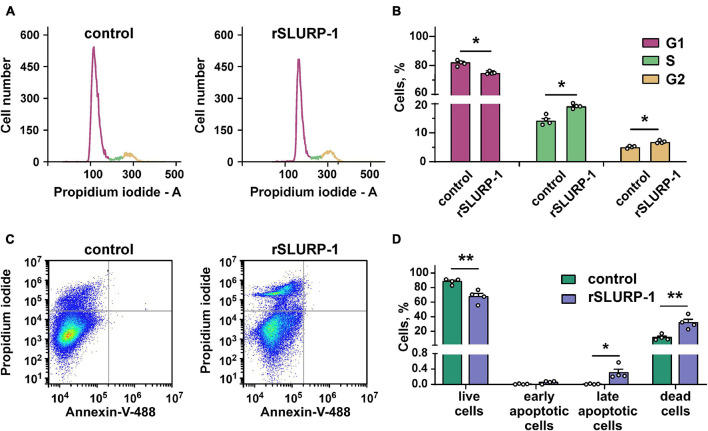
Influence of rSLURP-1 on cell cycle progression and apoptosis in A549 cells. **(A)** Representative nuclei population distributions of cells after 48 h incubation in absence (control) or presence of 1 μM rSLURP-1. **(B)** Percentage of cells in each cell cycle phase determined by ModFitLT software. Data are presented as mean ± SEM, *n* = 4; * (*p* < 0.05) indicates the significant difference from the control by two-tailed *t*-test. **(C)** Representative pictures of phosphatidylserine externalization analysis upon the rSLURP-1 treatment of A549 cells by flow cytometry with Annexin V-488 and Propidium iodide. The cells were incubated with 1 μM rSLURP-1 or without it (control) during 48 h. **(D)** Percentage of cells with externalized phosphatidylserine and bound propidium iodide. The data are presented as % of live, early apoptotic, late apoptotic and dead cells ± SEM (*n* = 4). * (*p* < 0.05) and ** (*p* < 0.01) indicate the significant difference from the control by two-tailed *t*-test.

Activation of the PI3K/AKT/mTOR signaling pathway is implicated in hyperproliferation and resistance to apoptosis (Mercurio et al., 2021). Since rSLURP-1 down-regulates this pathway, we studied its influence on cell cycle progression and apoptosis in A549 cells. Flow cytometry analysis revealed that rSLURP-1 induced significant reduction of A549 cell number in the G1 cell cycle phase from ∼81 to ∼74% with simultaneous increase of cell nuclei in the S and G2/M phases from ∼14 to ∼19% and from ∼5 to ∼7%, respectively (Figures 2A,B). The Annexin V phosphatidylserine externalization assay revealed significant increase in the number of non-apoptotically dead A549 cells, but did not reveal presence of apoptotic cells (Figures 2C,D). Thus, incubation of A549 cells with rSLURP-1 results in the cell cycle arrest in the S and G2/M phases and induces non-apoptotic cell death.

### rSLURP-1 Inhibits Migration of A549 Lung Cancer Cells but Not of Normal Lung Fibroblasts

Recombinant analogue of human SLURP-1 decreases the phosphorylation level of PDGFRβ (Y751) which could be associated with the receptor inhibition, while PDGFRβ activation leads to actin reorganization and cell migration (Heldin et al., 1998). Another protein, involved in the action of rSLURP-1, –mTOR, is also implicated in the control of cell motility, cytoskeleton assembly, and epithelial-mesenchymal transition (Zhou and Huang, 2011). In line with this, SLURP-1 has been shown to inhibit migration of pancreatic cancer cells (Throm et al., 2018).

**FIGURE 3:**
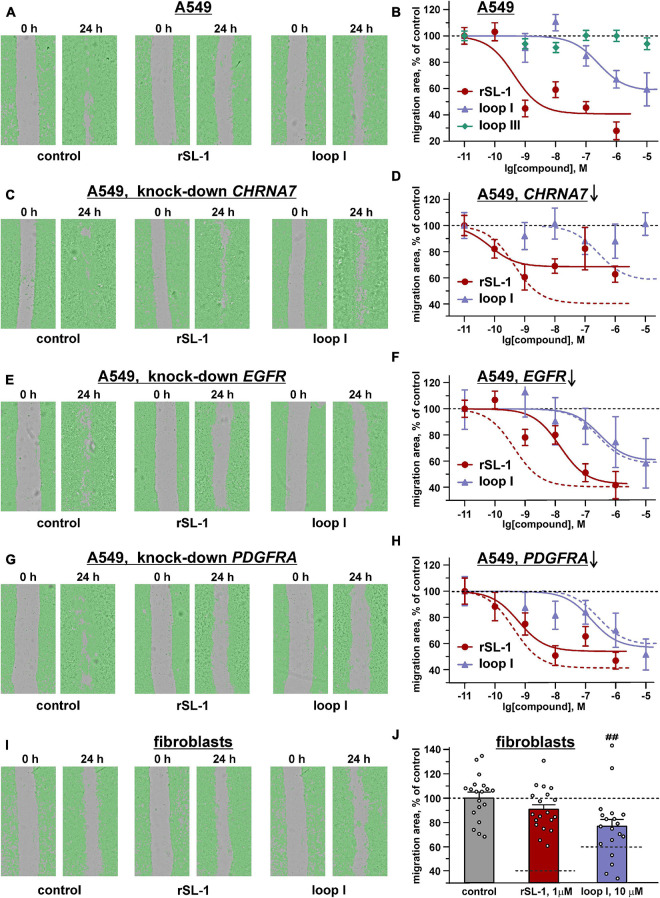
Influence of rSLURP-1 and the “loop I” and “loop III” peptides on migration of A549 cells **(A–H)** and primary mouse lung fibroblasts **(I,J)**. The data obtained for knockdown of the *CHRNA7*, *EGFR*, and *PDGFRA* genes are shown on the panels **(C,D)**, **(E,F)**, and **(G,H)**, respectively. **(A,C,E,G,I)** Representative pictures of scratch assay, obtained on CloneSelect Imager after 24 h incubation of the cells with 1 μM of rSLURP-1 or 10 μM of the “loop I” peptide. **(B,D,F,H,J)** Effect of different rSLURP-1 and the “loop I” and “loop III” peptides concentrations on the migration of the cells. Data are presented as a mean scratch surface, occupied by migrating cells (% normalized to control), ±SEM, *n* = 7–21; The parameters describing the dose-response curves (EC_50_, A_1_) and results of statistical comparisons are given in Table 1. Control level (100%) and position of the migration inhibition curves for rSLURP-1 and the “loop I” peptide in A549 cells without knock-down are shown by dashed lines. **(J)** Control level (100%) and the migration inhibition effects for 1 μM of rSLURP-1 and 10 μM of the “loop I” peptide in A549 cells are shown by dashed lines. ^##^ (*p* < 0.01) indicates significant difference from control group by one-way ANOVA/Dunnett’s test.

Therefore, we decided to test whether rSLURP-1 can inhibit migration of A549 cells. The scratch assay showed that rSLURP-1 significantly reduced migration of A549 cells up to ∼40% of the control with EC_50_ in nanomolar range (∼0.4 nM) (Figures 3A,B and Table 1). At the same time, rSLURP-1 at concentrations up to 1 μM did not affect the migration of primary lung fibroblasts (Figures 3I,J). Thus, rSLURP-1 showed selective anti-migration activity toward cancer cells.

**TABLE 1:** Parameters descripting the effect of *CHRNA7*, *EGFR*, and *PDGFRA* knock-down on the anti-migration activity of rSLURP-1 and synthetic peptide “loop I” in A549 cells^a^.

Gene knockdown	rSLURP-1		Peptide “loop I”	
	A_1_, %	EC_50_, nM	A_1_, %	EC_50_, nM
Control	40.8 ± 8.7	0.4 ± 0.2	58.7 ± 7.2	226 ± 85 (***)
α7-nAChR	68.5 ± 4.9 (#)	0.07 ± 0.04	n.d.	n.d.
EGFR	42.6 ± 8.7	14.1 ± 4.7 (###)	60.3 ± 8.6	291 ± 133
PDGFR	54.0 ± 4.9 (##)	0.6 ± 0.2	56.7 ± 9.9	155 ± 78

*^a^Data are presented as mean ± SEM, *n* = 7–21. ^∗∗∗^ (*p* < 0.001) indicates the significant difference between parameters describing rSLURP-1 and “loop I” curves by a two-tailed *t*-test. # (*p* < 0.05), ## (*p* < 0.01), and ### (*p* < 0.001) indicate the significant difference from parameters describing the control curve by a two-tailed *t*-test.*

We previously hypothesized that rSLURP-1 in A549 cells controls PTEN expression by acting on STAT3 (Shulepko et al., 2020b). There are other molecules that are involved in the regulation of gene expression,–small non-coding miRNAs (Catalanotto et al., 2016), and some of them controls genes related to migration (Xu et al., 2012; Othman and Nagoor, 2014; Smolarz and Widlak, 2021). Here, we tested the effect of rSLURP-1 on expression of these miRNA. No significant effect was found (Supplementary Figure 1). Therefore, the influence of rSLURP-1 on cell migration is not associated with the changes in expression of regulatory miRNAs.

### Loop I Is the Primary Site of SLURP-1 Responsible for Antiproliferative Activity

**FIGURE 4:**
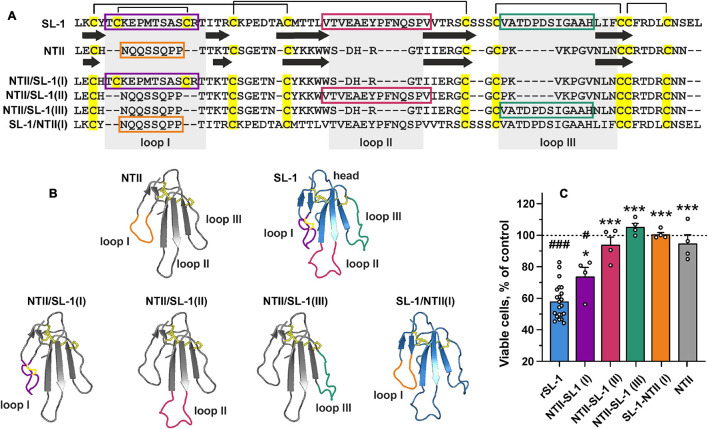
Design and study of antiproliferative activity of chimeric NTII/SL-1 and SL-1/NTII proteins. **(A)** Amino acid sequence alignment of SLURP-1, NTII, and chimeras. Cys residues are shown by yellow. Disulfide bonds are shown by brackets. The β-strands are designated by arrows. Sequences of the grafted loops are highlighted by color frames. **(B)** Schemes depicting the chimeric NTII/SL-1 and SL-1/NTII proteins. Color coding is the same as on the panel **(A)**. **(C)** Antiproliferative activity of the chimeric proteins in A549 cells according to WST-1 test. Cells were incubated during 48 h with 1 μM of the proteins. Data are presented as % of control (untreated cells, dashed line) ± SEM (*n* = 4–22). ^#^ (*p* < 0.05) and ^###^ (*p* < 0.001) indicate significant difference from the control by one-sample *t*-test. * (*p* < 0.05) and *** (*p* < 0.001) indicate significant difference from the “rSLURP-1” group by one-way ANOVA/Dunnett’s test.

Lymphocyte antigen 6/urokinase plasminogen activator receptor proteins have conservative spatial organization and contain the β-structural core stabilized by invariant disulfide bonds (“head”) and three elongated loops (“fingers”) protruding into solvent. Ly6/uPAR proteins usually interact with their targets by these loops (Vasilyeva et al., 2017). To identify the active site of SLURP-1, we constructed three chimeric proteins containing grafted loops (I, II, or III) of SLURP-1 on a scaffold of short three-finger neurotoxin II from cobra *Naja oxiana* (NTII) (Figures 4A,B). The chimeric proteins were named NTII/SL-1(I), NTII/SL-1(II), and NTII/SL-1(III). NTII was chosen as a scaffold, because it shares the conserved three-finger structure, but does not interact with α7-nAChRs (Lyukmanova et al., 2007) and does not inhibit the proliferation of A549 cells (Bychkov et al., 2019). Preservation of the overall three-finger spatial structure by NTII/SL-1 chimeras was confirmed by ^1^H NMR spectroscopy (Supplementary Figure 2). We have previously shown that rSLURP-1 inhibits A549 cell growth in a concentration-dependent manner and the maximum effect (∼60% of viable cells relative to the control) was observed at a concentration of 1 μM after 48 h incubation (Shulepko et al., 2020b). Here, we examined the antiproliferative activity of the chimeras in comparison with rSLURP-1 in A549 cells using a single concentration of 1 μM. The chimeras NTII/SL-1(II) and NTII/SL-1(III) and NTII itself were found to be completely inactive, while the chimera NTII/SL-1(I) containing the first loop of rSLURP-1 demonstrated antiproliferative activity slightly weaker than that of rSLURP-1 (inhibition effect up to ∼70% of viable cells relative to the control, Figure 4C). To further confirm the importance of the loop I region for rSLURP-1 activity, we designed an inverse chimera containing grafted loop I of NTII on a scaffold of rSLURP-1. As expected, the SL-1/NTII(I) chimera did not suppress growth of A549 cells (Figure 4C). Thus, the loop I region is the primary site of the SLURP-1 molecule responsible for antiproliferative activity in A549 cells.

### Synthetic Peptide Mimicking the Loop I of SLURP-1 Inhibits α7-nAChRs Expressed in *X. laevis* Oocytes

**FIGURE 5:**
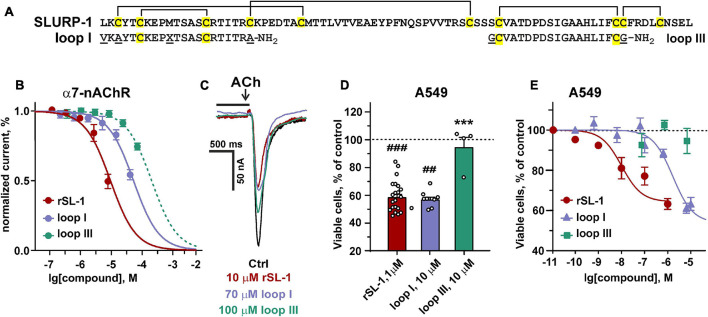
Design and activity of peptides, mimicking the SLURP-1 loops I and III. **(A)** Amino acid sequence alignment of rSLURP-1 and the synthetic peptides “loop I” and “loop III.” Cys residues are shown by yellow. Disulfide bonds are shown by brackets. **(B)** The dose-response curves for the inhibition of ACh-evoked currents at α7-nAChRs expressed in *X. laevis* oocytes by rSLURP-1 and synthetic peptides. The data are normalized to the peak amplitude of current recorded without compounds application (100%) and presented as mean ± SEM (*n* = 4–6 oocytes for each compound). The standard slope (nH = 1) inhibition equation was fitted to normalized data. **(C)** Representative responses to 100 ms pulses of 100 μM ACh recorded in absence and presence of rSLURP-1, “loop I” and “loop III” peptides. The period of oocyte pre-incubation with compounds is shown by horizontal bars above current traces (not in the timescale), the application of ACh is shown by arrow. **(D,E)** Antiproliferative activity of rSLURP-1, and “loop I” and “loop III” peptides in A549 cells upon 48 h incubation according to WST-1 test. Data are presented as % of control (untreated cells, dashed line) ± SEM (*n* = 4–22). ^##^ (*p* < 0.01) and ^###^ (*p* < 0.001) indicate significant difference from the control by one-sample *t*-test. *** (*p* < 0.001) indicates significant difference from the “rSLURP-1” group by one-way ANOVA/Dunnett’s test.

To further study the role of the loop I region in the SLURP-1 activity, we synthesized two peptide mimetics (Figure 5A). The first peptide (VKAYTCKEPXTSASCRTITRA-NH_2_, 21 residues, X stands for norleucine) mimics the structure of loop I of the SLURP-1 molecule. The second peptide (GCVATDPDSIGAAHLIFCG-NH_2_, 19 residues) has the sequence of loop III and was used as a negative control. Note, that the NTII/SL-1(III) chimera did not demonstrate antiproliferative activity in A549 cells (Figure 4C). Here and after the peptides are called as “loop I” and “loop III,” respectively (Figure 5A). Initially, we tested the activity of peptides in comparison with rSLURP-1 against α7-nAChRs expressed in *X. laevis* oocytes. In line with the previous findings (Lyukmanova et al., 2016a), rSLURP-1 and loop I demonstrated reversible concentration-dependent inhibition of α7-nAChRs with IC_50_ values of 12 ± 1 and 74 ± 4 μM, respectively. At the same time, loop III was much less active (IC_50_ > 250 μM, Figure 5B).

### The “Loop I” Peptide Suppresses Growth and Migration of A549 Cells but Not of Normal Fibroblasts

We have previously shown that rSLURP-1 suppresses the growth of various carcinomas, but demonstrates much weaker activity on the normal immortalized human cells, including keratinocytes Het-1A and lung fibroblasts WI-38 (Lyukmanova et al., 2016a, 2018; Shulepko et al., 2020b). Here, the antiproliferative and anti-migration activities of the “loop I” and “loop III” peptides were studied in comparison with rSLURP-1 in A549 cells or normal primary lung fibroblasts.

Water soluble tetrazolium salt 1 test revealed similar significant decrease of A549 cell viability upon treatment with 10 μM of “loop I” peptide or 1 μM of rSLURP-1 (Figure 5D). Comparison of the dose-response curves revealed EC_50_ of 1.8 ± 0.3 μM and 10.1 ± 2.6 nM for “loop I” and rSLURP-1, respectively (Figure 5E). Observed maximal effects were comparable (45.8 ± 5.1% and 59.0 ± 2.7% relative to the control for the “loop I” peptide and rSLURP-1, respectively), although the larger concentration of the “loop I” peptide was required (Figures 5D,E). The similar situation was observed for loop I in the scratch migration assay (Figures 3A,B). EC_50_ of the anti-migration activity was increased from 0.4 nM (rSLURP-1) to ∼230 nM (“loop I” peptide, Table 1), while the maximal effects were comparable (∼41 and 59%, respectively). Both the rSLURP-1 protein and “loop I” peptide demonstrated significantly diminished activity on normal fibroblasts in comparison with the cancer cells (Figures 3I,J). No effect of “loop III” on the A549 cell viability and migration was observed up to the 10 μM peptide concentration (Figures 3B, 5D,E).

### rSLURP-1 Forms Complexes With α7-nAChR and Receptor Tyrosine Kinases EGFR and PDGFRα

We recently proposed that some of the effects of rSLURP-1 in keratinocytes may be mediated by interaction with a second, different from α7-nAChR, molecular target (Shulepko et al., 2021). This second target may be some receptor of the RTK family, for example, EGFR forming a complex with α7-nAChR (Chernyavsky et al., 2015). On the other hand, the observed inhibition of PDGFRβ phosphorylation by rSLURP-1 (Figure 1) suggests an interaction of rSLURP-1 with PDGFRs.

**FIGURE 6:**
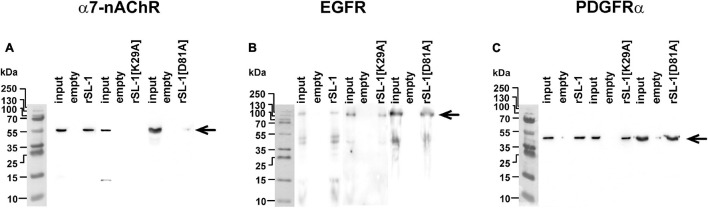
Analysis of rSLURP-1 targets in A549 cells. NHS-Sepharose coupled with rSLURP-1 or rSLURP-1[K29A] and rSLURP-1[D81A] mutants was incubated with a membrane fraction of A549 cells and extracted proteins were analyzed by Western blotting using antibodies against α7-nAChR **(A)**, EGFR **(B)**, and PDGFRα **(C)**. For detection of EGFR non-reducing SDS-PAGE was used. Lines: “Input”–the membrane fraction of A549 cells used for analysis; “empty”–proteins extracted from the membrane fraction by empty resin without rSLURP-1 or its mutant; “rSL-1”, “rSL-1[K29A]” or “rSL-1[D81A]”–proteins extracted from the membrane fraction by resin coupled with rSLURP-1 or its mutants. Bands corresponding to the α7-nAChR, EGFR, and PDGFRα receptors are shown by arrows.

In attempt to uncover this second target, we performed affinity purification of proteins from a membrane fraction of A549 cells that bind rSLURP-1. The NHS-Sepharose with coupled rSLURP-1 was used for affinity extraction; and resulting Western-blots were stained by antibodies to α7-nAChR, EGFR, and PDGFRα. Surprisingly, we found that rSLURP-1 extracts all three receptors from the membrane fraction (Figure 6). To test the specificity of the affinity extraction procedure, we used rSLURP-1 K29A mutant with a substitution in the loop I. According to the mutagenesis data, this mutation inactivates the rSLURP-1 protein and prevents the interaction with α7-nAChR (Shulepko et al., 2021). As expected, the K29A rSLURP-1 mutant did not extract α7-nAChR, while EGFR and PDGFRα did (Figure 6). Thus, loop I of SLURP-1 is responsible for the interaction with α7-nAChR, while some other parts of the molecule are involved in the interaction with EGFR and PDGFRα.

### rSLURP-1 and “Loop I” Antiproliferative Activity in A549 Cells Is Mediated Only by α7-nAChR, While in the Regulation of Migration α7-nAChR, EGFR, and PDGFRα Are Involved

**FIGURE 7:**
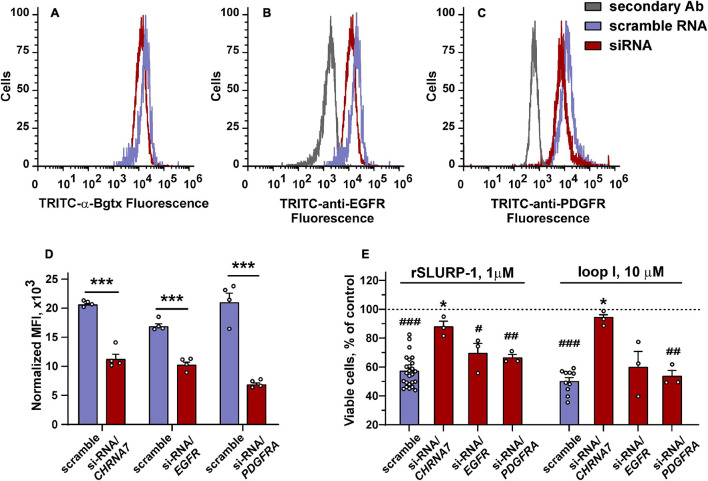
Influence of *CHRNA7*, *EGFR*, and *PDGFRA* knock-down on the rSLURP-1 and “loop I” peptide antiproliferative activity in A549 cells. **(A–C)** Representative histograms of the cell distribution after transfection with scramble RNA (purple) or siRNA to *CHRNA7*, *EGFR*, or *PDGFRA* (red) according to the intensity of TRITC-labeled α-Bgtx **(A)**, TRITC-labeled antibody against EGFR **(B)**, and TRITC-labeled antibody against PDGFRα **(C)**. **(D)** Median fluorescence intensities for TRITC-labeled α-Bgtx, TRITC-labeled antibody against EGFR, or TRITC-labeled antibody against PDGFRα in cells transfected with scramble RNA and in cells with the blocked α7-nAChR, EGFR, or PDGFRα expression (*n* = 4). ^∗∗∗^ (*p* < 0.001) indicates the significant difference between the groups by two-tailed *t*-test. **(E)** Influence of 1 μM rSLURP-1 or 10 μM “loop I” on proliferation of cells transfected with scramble RNA and in cells with blocked α7-nAChR, EGFR, or PDGFRα expression upon 48 h incubation. Data presented as % of control ± SEM (*n* = 3–25). ^#^ (*p* < 0.05), ^##^ (*p* < 0.01), and ^###^ (*p* < 0.001) indicate significant difference from control (untreated cells, dashed line) by one-sample *t*-test. ^∗^ (*p* < 0.05) indicates significant difference from the cells transfected with scramble RNA by one-way ANOVA/Dunnett’s hoc test.

To determine the role of α7-nAChR, EGFR, and PDGFRα in the rSLURP-1 effects, we knocked down the corresponding receptors genes, and studied the effect of rSLURP-1 and “loop I” on the proliferation and migration of A549 cells (Figures 7A–D). In line with the previous findings (Shulepko et al., 2020b), the antiproliferative activity of rSLURP-1 was almost completely abolished by the *CHRNA7* knockdown, while knockdown of the *EGFR* and *PDGFRA* genes did not affect the viability of A549 cells in the presence of rSLURP-1 (Figure 7E). Similarly, to rSLURP-1, the antiproliferative activity of the “loop I” peptide was inhibited only by the *CHRNA7* knockdown (Figure 7E).

The knock-down of the *CHRNA7* gene significantly diminished the maximal anti-migration effects of the rSLURP-1 protein and completely abolished the activity of the “loop I” peptide (Figures 3C,D and Table 1). Block of the *EGFR* expression did not influence the maximal amplitude of the anti-migration effect, while the EC_50_ value for rSLURP-1 became more than order of magnitude higher (increase from 0.4 to 14 nM, Figures 3E,F and Table 1). At the same time no effect on the activity of the “loop I” peptide was observed (Figures 3E,F). This indicates that EGFR is involved in the anti-migration effect of rSLURP-1, but not of the “loop I” peptide. Similarly, the knock-down of the *PDGFRA* gene did not affect the migration of A549 cells in the presence of the “loop I” peptide (Figures 3G,H), while significant reduction in the maximal effect amplitude, but not in EC_50_ value, was observed for rSLURP-1 (Figures 3G,H and Table 1). Taking together, the data obtained show that the anti-migration activity of the “loop I” peptide depends only on the α7 nicotinic receptor, while the activity of rSLURP-1 depends on the α7-nAChR and RTKs (EGFR and PDGFRα).

## Discussion

Activation of α7-nAChRs by nicotine results in increase of viability, proliferation, and motility of epithelial cells (Lupacchini et al., 2020). Using the lung adenocarcinoma A549 cells, for the first time we showed that rSLURP-1 significantly reduced not only proliferation of cancer cells, but also their migration (Figure 3),–the process that underlies tumor invasion and metastasis. The opposite effect of rSLURP-1 to that of nicotine is in good agreement with its negative modulation of the α7 receptor (Lyukmanova et al., 2016a). Notably, very weak rSLURP-1 effect was detected in normal cells (primary mouse fibroblasts), pointing on existence of so called “pharmacological window,” similar to that found for antiproliferative effect on normal keratinocytes (Lyukmanova et al., 2018).

Recently, we proposed the mutagenesis-based model of the α7-nAChR/SLURP-1 complex, according which loops I, II, and III of SLURP-1 form multiple contacts with the receptor (Shulepko et al., 2021). Loop I was proposed to interact with the loop C of the primary receptor’s subunit. The loop C covers the orthosteric ligand binding site of the nicotinic receptor, and its movement is coupled with the receptor activation (Law et al., 2005; Gulsevin, 2020). Loop II and loop III of SLURP-1 were supposed to form additional contacts with the primary and complementary receptor subunits (Shulepko et al., 2021). The data obtained here revealed the primary role of loop I of the SLURP-1 molecule for the interaction with α7-nAChR (Figures 4, 5). Accordingly, the K29A mutation in loop I resulted in a decrease in the affinity of rSLURP-1 toward α7-nAChR in A549 cells (Figure 6A) as it has been demonstrated in keratinocytes (Shulepko et al., 2021). The reduced activity of the “loop I” peptide, as compared to the whole rSLURP-1 molecule, confirms the multiple point mode of the α7-nAChR/SLURP-1 interaction proposed earlier (Shulepko et al., 2021). In line with this, we observed the weak inhibitory activity of the “loop III” peptide at α7-nAChRs expressed in oocytes and reduced affinity of the mutant with substitution D81A in loop III toward α7-nAChR (Figures 5C, 6A). This indicates that loop III also plays some role in the interaction with the receptor.

Notably, the mode of the α7-nAChR/SLURP-1 interaction differs from that found for other Ly6/uPAR proteins and their targets. For example, the region of loop II was found to be important for interaction of mambalgins with the acid sensitive ion channel ASIC1 (Salinas et al., 2021), for fasciculins with acetylcholinesterase (Falkenstein and Pena, 1997), for α-cobratoxin with the GABA_*A*_ receptor (Kudryavtsev et al., 2015), as well as for human neuromodulator Ly6/neurotoxin 1 (Lynx1) with α7-nAChR (Lyukmanova et al., 2013). Loop II frequently contains almost all the determinants necessary for efficient interaction of snake α-neurotoxins with the nicotinic receptors (Lyukmanova et al., 2007, 2016b; Rahman et al., 2020). Similarly to the α7-nAChR/SLURP-1 complex, the α-neurotoxins mainly interact with the loop C of the nicotinic receptors.

Early reports have described SLURP-1 as a selective ligand of α7-nAChR (Lyukmanova et al., 2016a). However, recently we have proposed that, except α7-nAChR, SLURP-1 can also interact with other receptors, probably from the RTK family (Shulepko et al., 2021). It has been suggested, that the site responsible for the interaction with RTK(s) is located in the “head” region of the SLURP-1 molecule. The hypothesis about the SLURP-1/RTK interaction was also supported by dependence of the SLURP-1-induced inhibition of A549 cell growth simultaneously on α7-nAChR and EGFR (Shulepko et al., 2020b). Moreover, SLURP-1-induced pro-inflammatory cytokine secretion by mast cells was found to be not connected with α7-nAChR, that also points on the existence of other targets of SLURP-1 (Ertle et al., 2021). Here, we also found some evidences to support this hypothesis. One of them is given by the migration scratch assay in A549 cells, where the knock-down of *CHRNA7* gene does not completely abolish the anti-migration effect of rSLURP-1 (Figures 3C,D).

The data mentioned above imply the presence of alternative mechanisms of the antiproliferative and anti-migration activity of SLURP-1 mediated by other (non α7-nAChR) receptor(s). In line with this assumption, rSLURP-1 extracted not only α7-nAChR, but also EGFR and PDGFRα from the A549 cell membrane fraction (Figures 6B,C). The involvement of these receptors in the anti-migration effect of SLURP-1 was confirmed by knock-down experiments (Figures 3E–H). Knock-down of the *EGFR* or *PDGFRA* genes diminished EC_50_ or maximal amplitude of the rSLURP-1 effect, respectively. Thus, anti-migration activity of SLURP-1 in A549 cells depends on all three receptors: α7-nAChR, EGFR, and PDGFRα (Figure 3). Interestingly, the effect of knock-down on the antiproliferative activity in A549 cells was different (Figure 7E). In this case, the *CHRNA7* knock-down almost completely abolished the rSLURP-1 antiproliferative effect, while knock-down of the *EGFR* or *PDGFRA* genes did not influence the maximal amplitude of the effect. This finding illustrates the difference in the molecular mechanisms that control proliferation and migration of A549 cells. Interestingly, the *CHRNA7* knock-down led to complete cancelation of the antiproliferative and anti-migration effect of the “loop I” peptide (Figures 3, 5E) indicating that α7-nAChR is the only target of this peptide. Apparently, rSLURP-1 interacts with EGFR and PDGFRα not by loop I, but by other parts of the molecule as it was proposed earlier (Shulepko et al., 2021).

Participation of two different RTKs in the SLURP-1 activity may look quite surprising. Two possibilities can be considered to explain it: simultaneous formation of two types of the complexes, –α7-nAChR/EGFR and α7-nAChR/PDGFR, both of which can bind SLURP-1, or formation of a complex between α7-nAChR and the EGFR/PDGFR heterodimer. Previously, the direct interaction of α7-nAChR with EGFR (Chernyavsky et al., 2015) and the possible formation of functional heterodimeric complexes EGFR/PDGFR (Saito et al., 2001; Perrone et al., 2009; Chakravarty et al., 2017) were reported. Thus, regardless of choice of the correct model, we have shown here for the first time that complexes of RTK and α7-nAChR can interact with a common modulator.

The PI3K/AKT/mTOR pathway is known to regulate growth, migration, and drug resistance in A549 cells (Wang et al., 2020; Morelli et al., 2021), Recently we have proposed that rSLURP-1 regulates this pathway by increasing expression of PTEN, –the negative regulator of PI3K (Shulepko et al., 2020b). In line with this hypothesis, here we observed that SLURP-1 induces significant inhibition of mTOR (S2448) and PTEN (S380) phosphorylation (Figure 1), which corresponds to inactivation of mTOR and activation of PTEN (Scully et al., 2014). The observed activation of PTEN on a functional level can explain the reduction of A549 cell growth and migration by rSLURP-1. Interestingly, we did not observe the changes in phosphorylation of the key kinases from other intracellular pathways (ERK and p38 mitogen-activated protein kinase, Figure 1), indicating that the effects of SLURP-1 in A549 cells are probably not mediated by these pathways. This is in a good agreement with the data of the inhibitory analysis showed that these pathways are not involved in the SLURP-1 action (Shulepko et al., 2020b).

Phosphoinositide 3-kinase is one the key players of the PI3K/AKT/mTOR signaling pathway (Porta et al., 2014). It was shown previously, that α7-nAChR can form a complex with PI3K in lung cancer cells (Chernyavsky et al., 2015). From the other hand, the direct interaction of PI3K with EGFR and PDGFR was reported (Valius et al., 1993; Klinghoffer et al., 1996; Kharbanda et al., 2020). In addition, we also found the weak insignificant decrease in phosphorylation of non-receptor tyrosine kinase Src at position Y416 under action of SLURP-1 (Figure 1), corresponding to inactivation of this kinase (Irtegun et al., 2013). The Src kinase can be directly activated by various RTKs (including EGFR and PDGFR) (Takikita-Suzuki et al., 2003; Furcht et al., 2015) and, in turn, induces activation of the PI3K kinase and STAT3 transcription factor (Byers et al., 2009; Beadnell et al., 2018). In line with that, the involvement of STAT3 in the action of SLURP-1 on A549 cells was previously shown by inhibitory analysis (Shulepko et al., 2020b).

Induction of the cell cycle arrest in the S and G2/M phases in A549 cells upon incubation with rSLURP-1 (Figures 2B,C) is consistent with the fact that activation of the PI3K/AKT/mTOR signaling pathway is required to overcome G2/M cell cycle checkpoint (Kandel et al., 2002). Indeed, partial deficiency in the mTOR activity is sufficient to block cells from G2/M checkpoint recovery (Hsieh et al., 2018). Thus, inhibition of the PI3K/AKT/mTOR pathway components, including mTOR, by rSLURP-1 may attenuate cell cycle progression and lead to the accumulation of cells in the G2/M phase.

Extensive oxidative stress or Ca^2+^ excess in the cytoplasm can cause the mitochondrial membrane permeabilization without release of apoptotic molecules, which in turn can drive necrotic cell death rather than apoptosis (Shaheen et al., 2011). Previously, we showed that IP3 receptors regulating Ca^2+^ release from the endoplasmic reticulum are implicated in the antiproliferative action of rSLURP-1 in A549 cells (Shulepko et al., 2020b). Thus, absence of apoptosis in A549 cells upon the rSLURP-1 treatment (Figures 2C,D) can be linked with excessive Ca^2+^ release from the intracellular depot and could point on induction of non-apoptotic cell death such as necroptosis or necrosis.

Besides SLURP-1, lung cancer cells express Lynx1,–another Ly6/uPAR protein, also involved in control of their growth (Fu et al., 2015; Bychkov et al., 2019). In spite of the common target for both proteins in A549 cells (α7-nAChR), there are many differences in the molecular mechanisms underlying the Lynx1 and SLURP-1 action. In contrast to SLURP-1, Lynx1 induces the cell cycle arrest in the G0/1 and G2/M phases in A549 cells after 24 and 72 h incubation, respectively, caused apoptosis, and its action is described by the two-stage mechanism involving both AKT, ERK, and other mitogenic pathways (Bychkov et al., 2019). Such differences in action may be explained by the fact that Lynx1, not like SLURP-1, is the GPI-anchored protein (Miwa et al., 1999), and simultaneous interaction of membrane-anchored Lynx1 with α7-nAChR, and RTKs is sterically impossible.

**FIGURE 8:**
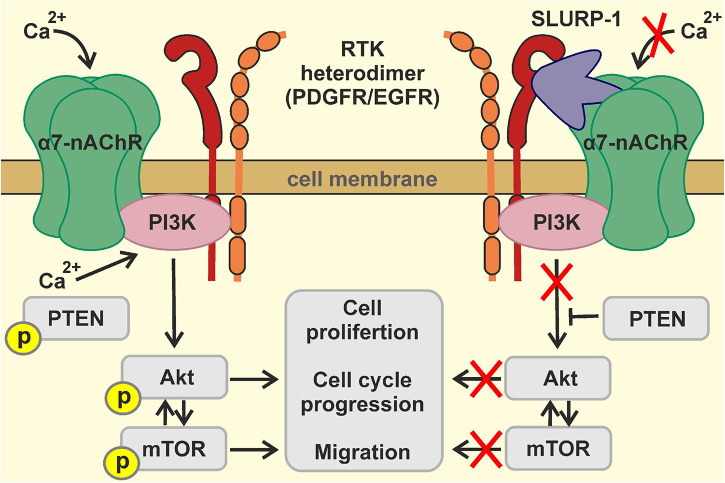
Scheme illustrating the mechanism of SLURP-1 action in A549 cells.

Based on all obtained data, we can propose the following mechanism of the SLURP-1 action (Figure 8): binding of SLURP-1 to the complex α7-nAChR/EGFR/PDGFR results in reduction of phosphorylation of PDGFR at the Y751 site; this in turn diminishes phosphorylation of PI3K. Together with the mTOR inactivation and activation of PTEN this leads to inhibition of the PI3K/AKT/mTOR signaling pathway resulting in down-regulation of proliferation and migration of lung cancer cells, cell cycle arrest, and non-apoptotic cell death.

In summary, antiproliferative activity of SLURP-1 in lung cancer cells is mainly mediated by interaction with α7-nAChR, which is realized by means of loop I. However, other molecular targets, e.g., EGFR and PDGFR, are involved in control of lung cancer cell migration by SLURP-1. This multi-targeted interaction leads to inhibition of the PI3K/AKT/mTOR signaling. Despite of lower activity, the synthetic peptide “loop I” demonstrates many common properties with rSLURP-1 such as inhibition of proliferation and migration of cancer cells, reversibility of interaction with α7-nAChR, and reduced activity on normal lung fibroblasts (Figure 5). Thus, synthetic “loop I” is a promising prototype of new antitumor drug with the properties close to the native SLURP-1 protein.

## Data Availability Statement

The original contributions presented in the study are included in the article/Supplementary Material, further inquiries can be directed to the corresponding author/s.

## Ethics Statement

The animal study was reviewed and approved by IBCH RAS IACUC, protocol #312 from 21 December 2020.

## Author Contributions

MS, MB, ZS, and EL: conceptualization and methodology. MB and EL: data curation. MB, MS, and EL: formal analysis. MS, EL, and MK: funding acquisition. MS, MB, OS, AP, DK, SK, VA, IC, and LB: investigation. MS, ZS, EL, and MK: project administration. ZS, EL, and MK: resources. MB and MS: software and writing—original draft preparation. ZS and EL: supervision and writing—review and editing. MS, MB, ZS, EL, and AP: visualization. All authors contributed to the article and approved the submitted version.

## Conflict of Interest

The authors declare that the research was conducted in the absence of any commercial or financial relationships that could be construed as a potential conflict of interest.

## Publisher’s Note

All claims expressed in this article are solely those of the authors and do not necessarily represent those of their affiliated organizations, or those of the publisher, the editors and the reviewers. Any product that may be evaluated in this article, or claim that may be made by its manufacturer, is not guaranteed or endorsed by the publisher.

## Supplementary Material

Supplementary Figure S1 Analysis of expression of miRNa implicated in A549 cell migration by qPCR. Cells were treated with 1 μM rSLURP-1 for 24 h, total RNA was isolated and stem-loop primers were used for cDNA synthesis. After that qPCR with specific primers (Supplementary Table 2) was performed. The miRNA expression levels are normalized to U6 non-coding RNA level.

Supplementary Figure S2 Fragments of 1D ^1^H NMR-spectra of rSLURP-1, NTII, and their chimeras (700 MHz, 30°C). Amide protons regions of the spectra are shown.

Supplementary Figure 3 Characterization of the synthetic loop I and loop III. Analysis of synthetic “loop I” **(A,B,C)** and synthetic “loop III” **(D,E,F)** by HPLC, MALDI, and ^1^H-NMR spectroscopy is shown.

Supplementary Table 1 The siRNA sequences for knock-down of the *CHRNA7, EGFR*, and *PDGFRA* genes.

Supplementary Table 2 The primers used for analysis of miRNA expression.
