# Supplementary material for: SLURP-1 Controls Growth and Migration of Lung Adenocarcinoma Cells, Forming a Complex With α7-nAChR and PDGFR/EGFR Heterodimer
Source: Front Cell Dev Biol. 2021 Sep 14;9:739391. doi: 10.3389/fcell.2021.739391 (PMC8476798; doi:10.3389/fcell.2021.739391)
Supplement: Supplementary Figure S1 — Analysis of expression of miRNa implicated in A549 cell migration by qPCR. Cells were treated with 1 μM rSLURP-1 for 24 h, total RNA was isolated and stem-loop primers were used for cDNA synthesis. After that qPCR with specific primers (Supplementary Table 2) was performed. The miRNA expression levels are normalized to U6 non-coding RNA level. [file Data_Sheet_1.docx]

SUPPLEMENTAL INFORMATION

SLURP-1 controls growth and migration of lung adenocarcinoma cells, forming a complex with α7-nAChR and PDGFR/EGFR heterodimer

Maxim L. Bychkov^1,#^, Mikhail A. Shulepko^1,#^, Olga V. Shlepova^1,2^, Dmitry S Kulbatskii^1^, Irina A. Chulina^3^, Alexander Paramonov^4^, Ludmila K. Baidakova^3^, Viatcheslav N. Azev^3^, Sergei G. Koshelev^1^, Mikhail P. Kirpichnikov^1,5^, Zakhar O. Shenkarev^2,4^, Ekaterina N. Lyukmanova^1,2,5^*

^1^Shemyakin-Ovchinnikov Institute of Bioorganic Chemistry RAS, Protein Engineering Department, Moscow, Russia

^2^Moscow Institute of Physics and Technology, Phystech School of Biological and Medical Physics, Dolgoprudny, Moscow Region, Russia

^3^Branch of Shemyakin-Ovchinnikov Institute of Bioorganic Chemistry RAS, Group of Peptide Chemistry, Pushchino, Moscow Region, Russia

^4^Shemyakin-Ovchinnikov Institute of Bioorganic Chemistry RAS, Structural Biology Department, Moscow, Russia

^5^Lomonosov Moscow State University, Biological Faculty, Moscow, Russia

^#^These authors made equal contribution

***Correspondence:**Ekaterina N. Lyukmanova,
[ekaterina-lyukmanova@yandex.ru](mailto:ekaterina-lyukmanova@yandex.ru)

**Table S1. The siRNA sequences for knock-down of the *CHRNA7*, *EGFR*, and *PDGFRA genes.***

| **Gene** | **siRNA sequence** | |
| --- | --- | --- |
|  | **sense** | **antisense** |
| *CHRNA7* | UAAAAACAUCCUUAGUAAGAC | CUUACUAAGGAUGUUUUUACC |
|  | AAAUGUUGGUGGUUAAAACUU | GUUUUAACCACCAACAUUUGG |
|  | UCAUUAAAGAAUAAGAAUGAG | CAUUCUUAUUCUUUAAUGAGA |
| *EGFR* | UGAUCUGUCACCACAUAAUUACGG | CCCGUAAUUAUGUGGUGACAGAUCA |
|  | UUAGAUAAGACUGCUAAGGCAUAGG | CCUAUGCCUUAGCAGUCUUAUCUAA |
|  | UUUAAAUUCACCAAUACCUAUUCCG | CGGAAUAGGUAUUGGUGAAUUUAAA |
| *PDGFRA* | GUGGCCAUUAUACUAUUGU | ACAAUAGUAUAAUGGCCAC |
|  | GGCCACAUUUGAACAUUGU | ACAAUGUUCAAAUGUGGCC |
|  | GCUGCCUGGACAAUAUAAA | UUUAUAUUGUCCAGGCAGC |
| Scramble | UUCUCCGAACGUGUCACGU | ACGUGACACGUUCGGAGAA |

**Table S2. The primers used for analysis of miRNA expression**

| **miRNA** | **Stem-loop primer** | **Forward primer** | **Reverse primer** |
| --- | --- | --- | --- |
| ***U6 snRNA*** | CGCTTCACGAATTTGCGTGTCA | GCTTCGGCAGCACATATACTAAAAT | CGCTTCACGAATTTGCGTGTCAT |
| ***miR-7*** | CACCGTTCCCCGCCGTCGGTGACAACA | CGCCCTGGAAGACTAGTGAT | CCGTCGGTGACAACAAAAT |
| ***miR-21*** | GTCGTATCCAGTGCAGGGTCCGAGGTATTCGCACTGGATACGACTCAACA | GCCCGCTAGCTTATCAGACTGATG | CAGTGCAGGGTCCGAGGT |
| ***miR-31*** | GTCGTATCCAGTGCAGGGTCCGAGGTATTCGCACTGGATACGACAGCTAT | GCCGCAGGCAAGATGCTGGC |  |
| ***miR-96*** | GTCGTATCCAGTGCAGGGTCCGAGGTATTCGCACTGGATACGACAGCAAA | GCCCGCTTTGGCACTAGCACATT |  |
| ***miR-135b*** | GTCGTATCCAGTGCAGGGTCCGAGGTATTCGCACTGGATACGACTCACAT | GCCCGCTATGGCTTTCATTCCT |  |
| ***miR-203*** | GTCGTATCCAGTGCAGGGTCCGAGGTATTCGCACTGGATACGACCTAGTGGTC | GTATCCAGTGCAGGGTCCGA | CGACGGTGAAATGTTTAG |
| ***miR-221*** | CACCGTTCCCCGCCGTCGGTGGAAACC | CGGGCAGCTACATTGTCTG | CGTCGGTGGAAACCAGCA |
| ***miR-451*** | CACGGAACCCCGCCGACCGTGAACTCA | CGCCGAAACCGTTACCAT | GCCGACCGTGAACTCAGTAAT |


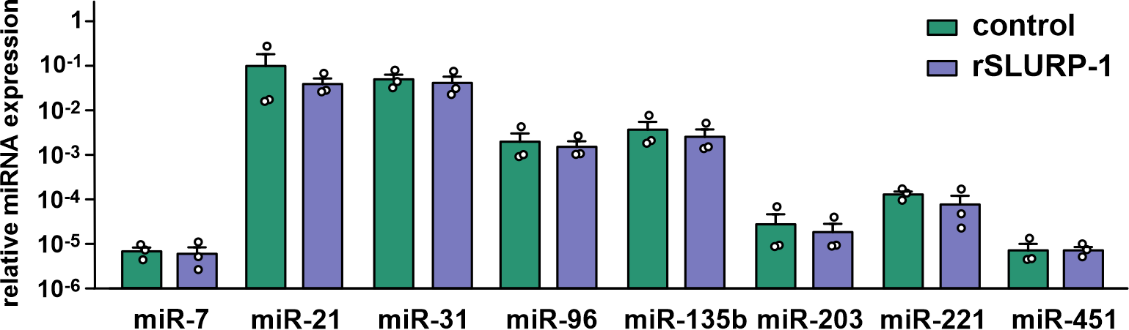


**Figure S1. Analysis of expression of miRNa implicated in A549 cell migration by qPCR.** Cells were treated with 1 µM rSLURP-1 for 24 h, total RNA was isolated and stem-loop primers were used for cDNA synthesis. After that qPCR with specific primers (Table S2) was performed. The miRNA expression levels are normalized to U6 non-coding RNA level.


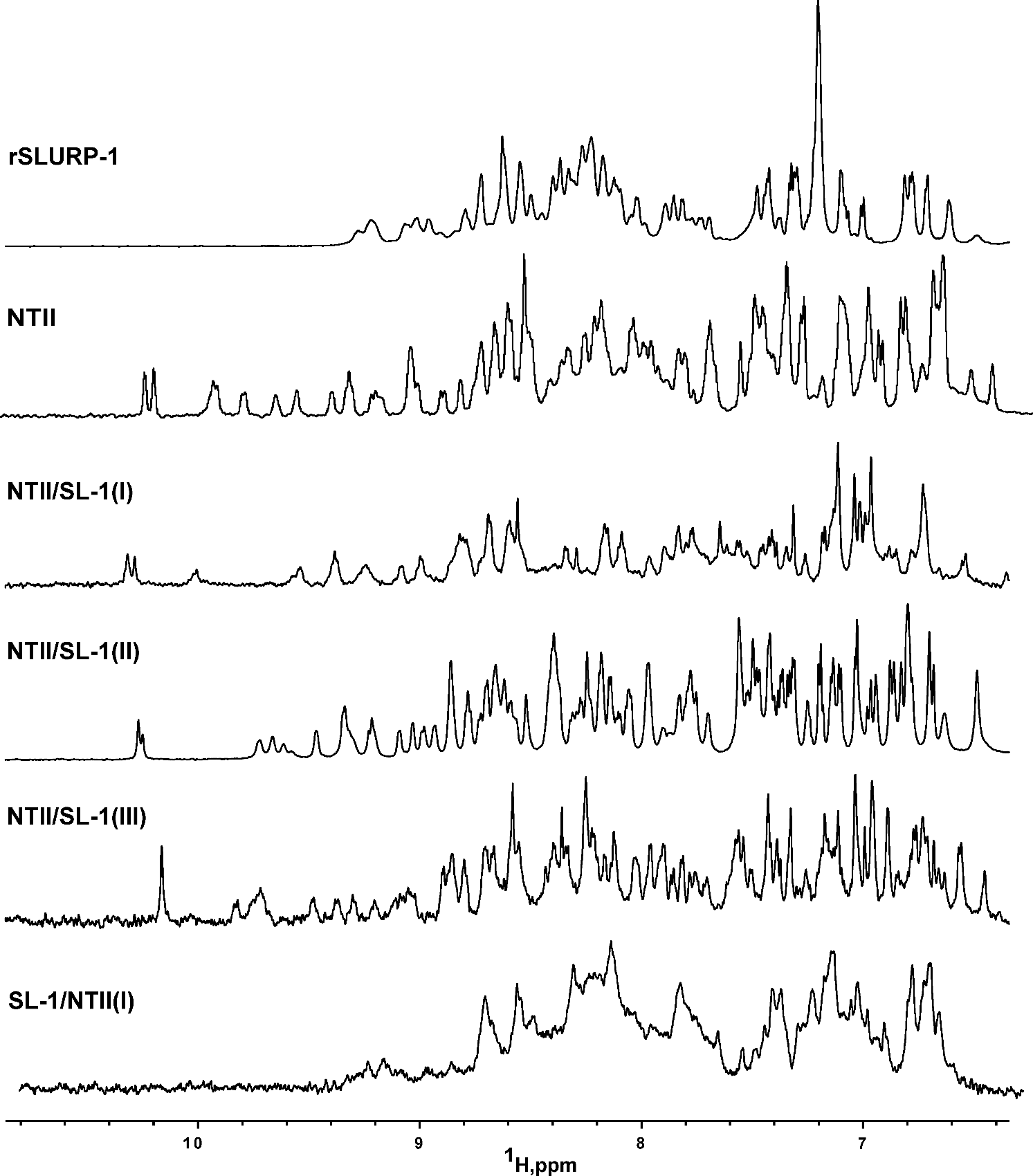


**Figure S2.** Fragments of 1D ^1^H NMR-spectra of rSLURP-1, NTII, and their chimeras (700MHz, 30ºC). Amide protons regions of the spectra are shown.

| 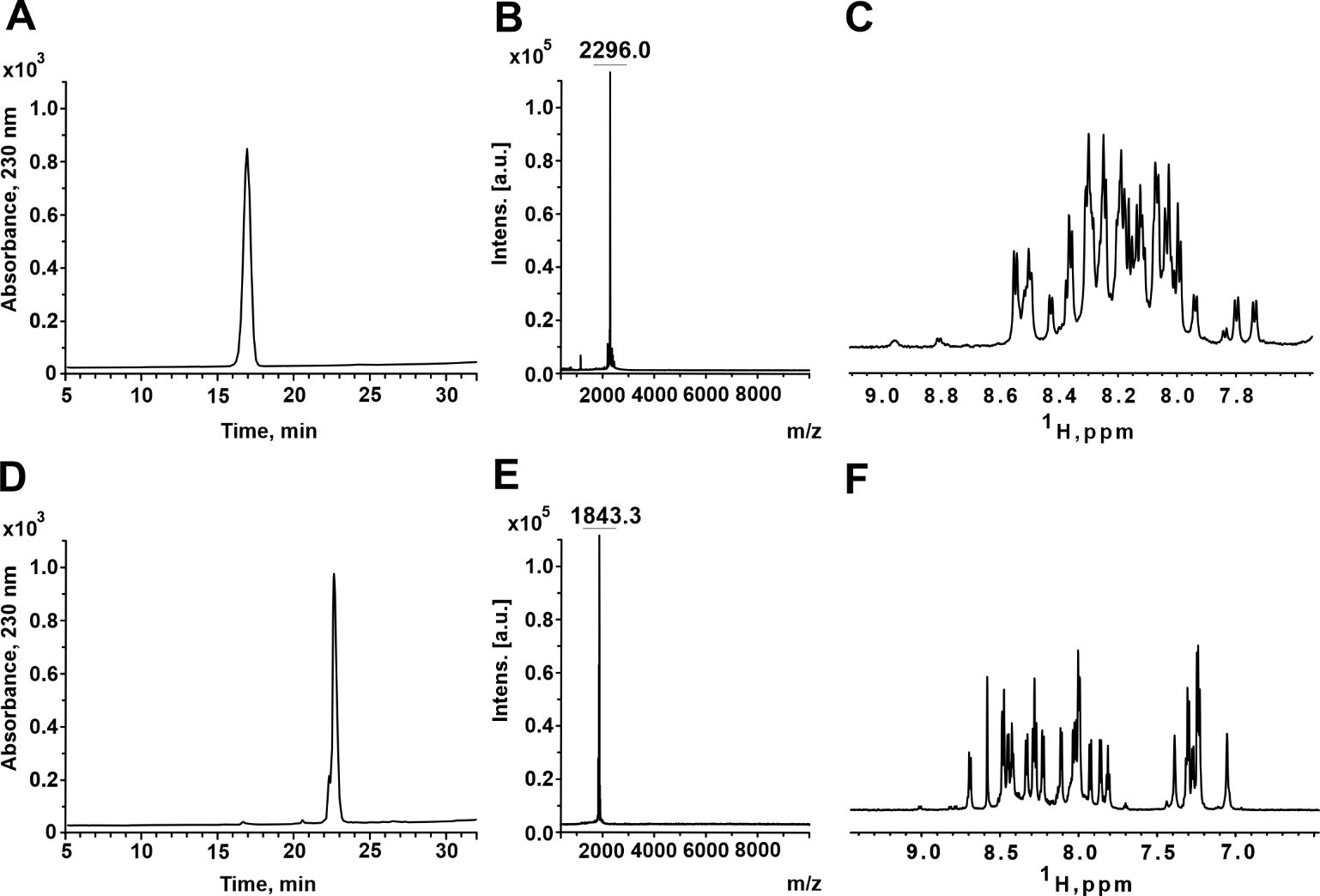 |
| --- |
| **Figure S3. Characterization of the synthetic loop I and loop III.** Analysis of synthetic ‘loop I’ (A,B,C) and synthetic ‘loop III’ (D,E,F) by HPLC, MALDI, and ^1^H-NMR spectroscopy is shown. |
